# WHO Tiered-Effectiveness Counseling Is Rights-Based Family Planning

**DOI:** 10.9745/GHSP-D-15-00096

**Published:** 2015-08-12

**Authors:** John Stanback, Markus Steiner, Laneta Dorflinger, Julie Solo, Willard Cates

**Affiliations:** ^a^​ FHI 360, Durham, NC, USA; ^b^​ Independent Consultant, Durham, NC, USA

## Abstract

Contraceptive effectiveness is the leading characteristic for most women when choosing a method, but they often are not well informed about effectiveness of methods. Because of the serious consequences of “misinformed choice,” counseling should proactively discuss the most effective methods—long-acting reversible contraceptives and permanent methods—using the WHO tiered-effectiveness model.

Improving access to long-acting, reversible methods of contraception (LARCs)—which provide highly effective, long-term, and easy-to-use protection against unintended pregnancy—is of crucial importance to the lives of countless individual women. Yet, among family planning experts, consensus remains elusive on the important issue of whether and how client counseling should emphasize this top tier of highly effective contraceptive methods. Research consistently shows women believe effectiveness is one of the most important factors—usually *the* most important factor—when choosing a contraceptive method,^1^ but accurate knowledge of contraceptive effectiveness remains poor.^2^

In this paper, we argue for proactive counseling based on the World Health Organization (WHO) tiered-effectiveness chart that begins with the relative effectiveness of various methods as a way to provide truly informed choice. All contraceptives are not created equal. Counseling that does not focus on effectiveness can lead to “misinformed choice,” which may undermine rights-based approaches.

## RIGHTS-BASED FAMILY PLANNING

Recent WHO guidance, summarizing findings of a technical consensus meeting on contraceptive choice and human rights, advises programs on how to ensure human rights are respected and protected when services are scaled-up to reduce unmet need for family planning.^3^ This seminal document was reinforced and extended through a new conceptual framework for human rights-based family planning.^4^^,^^5^

The guidance was in part a reaction to civil society concerns about the ambitious, numerical goals of the Family Planning 2020 (FP2020) initiative, launched in London in 2012, which aims to extend modern contraceptive access to 120 million additional clients by 2020.^6^ In addition, this new series of publications also builds upon the international family planning community’s nearly 50-year history of commitment to voluntarism and promoting clients’ right to free choice in family planning.^3^ This rights-based message was affirmed at international population conferences such as the landmark International Conference on Population and Development in Cairo in 1994 and in a variety of reproductive health and rights frameworks adopted by normative bodies before and since.

Taken together, the new documents about rights-based family planning are an important reminder of the need for voluntary, coercion-free contraceptive services. Those committed to the sexual and reproductive health field should have these values at their core.

## IMPACT OF LARCS

The renewed focus on rights-based family planning coincides with emerging findings from a variety of recent programs in both developed^7^^,^^8^ and developing^9^^-^^13^ countries to increase access to LARCs. In the most well known of these, the St. Louis CHOICE study, thousands of women were offered free, same-day contraceptive services and followed for up to 3 years to document reproductive health and other outcomes.^7^ Given availability of free contraception, 70% to 75% of women (including teens) chose LARCs,^14^^,^^15^ and both continuation and satisfaction were significantly higher among LARC users than non-LARC users.^16^ Non-LARC users in St. Louis were 20 times more likely to become pregnant in the next 3 years than LARC users.^14^ The powerful results from CHOICE have been a clarion call for the importance of making LARCs widely available in family planning programs.

In the large CHOICE study, in which 70%–75% of women chose LARCs, continuation and satisfaction were higher, and pregnancy rates much lower, among LARC vs. non-LARC users.

**FIGURE 1:**
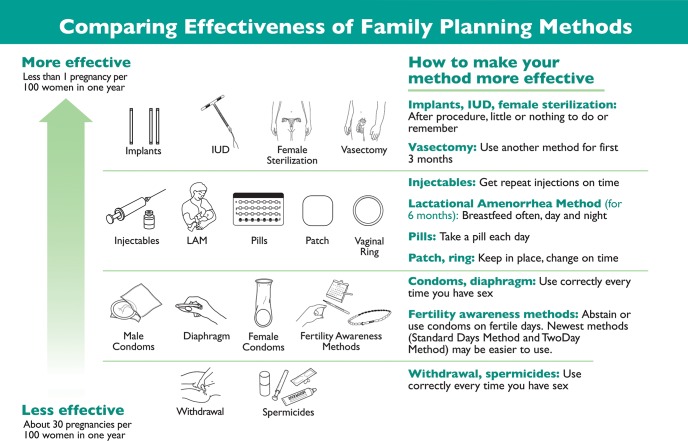
World Health Organization Model of Tiered Contraceptive Effectiveness Source: Steiner et al.,^17^ Trussell,^18^ and WHO.^19^

The CHOICE program offered a wide variety of methods and actively counseled clients about methods using the gradient of WHO’s “tiered” contraceptive effectiveness chart (Figure 1). Trained counselors described the full range of contraceptive methods and used internationally accepted counseling methods and tools, such as GATHER,^20^ to provide personalized counseling based on clients’ reproductive health needs.^21^ Within this framework of client-centered counseling, providers proactively spoke first about and emphasized the LARC methods of intrauterine devices (IUDs) and implants in the highest tier of contraceptive effectiveness. This counseling approach was dubbed “LARC-first.”^22^

## BALANCING REPRODUCTIVE JUSTICE AND TIERED COUNSELING

While the response to these programs has been overwhelmingly positive, some observers have begun a constructive dialogue about the potential pitfalls of embracing “LARC-first” without also emphasizing the necessary rights-based framework.^23^^-^^25^ They rightfully caution that in some situations tiered counseling could become too directive, or perhaps even coercive. This is especially worrisome in dealing with more vulnerable populations. We agree with these authors that the answer lies in striking a “delicate balance.”^25^ In particular, we support the position that “reproductive justice would enable women to access and use LARC if they wish to, but also to dispense with LARC and/or have LARC methods removed if they wish to.”^24^

Providers must strike a delicate balance between embracing “LARC-first” counseling while emphasizing reproductive rights.

Clinicians should not and, indeed, have no need to “push” LARCs. Evidence shows that when LARCs are available and affordable, most clients, if fully informed about effectiveness and relevant method characteristics, will choose them of their own accord.^7^^-^^11^ On the other hand, we disagree with the notion that other method characteristics should *a priori* be on par with that of effectiveness. In our view, the effectiveness of any contraceptive method is its paramount characteristic, and counseling that does *not* use WHO tiers (with the most effective methods discussed proactively) fails to meets the true needs and desires of the majority of women.

## OUR PREMISES: CLIENT AUTONOMY, SAFETY, AND ACCURATE INFORMATION

We take as given the new WHO guidance (excerpted below), highlighting the paramount importance of client autonomy in decision making^3(p.19)^:

*Respecting autonomy in decision-making requires that any counselling, advice or information that is provided by health workers or other support staff should be non-directive, enabling individuals to make decisions that are best for themselves. People should be able to choose their preferred method of contraception, taking into consideration their own health and social needs*.

We also take safety as a given, assuming that the global regulatory framework properly ensures the safety of modern contraceptives and, for the purpose of this paper, that the appropriate methods are safely provided to, and safely used by, clients.

Finally, we assume a right to non-directive counseling that conveys accurate information, including information about effectiveness and likelihood of pregnancy. This right is also emphasized in the new WHO guidance (excerpted below),^3(p.19)^ as well as in earlier international conventions and covenants^26^^,^^27^:

*Individuals have the right to be fully informed by appropriately trained personnel. Health-care providers have the responsibility to convey accurate, clear information, using language and methods that can be readily understood by the client, together with proper, non-coercive counselling, in order to facilitate full, free and informed decision-making. … The information provided to people so that they can make an informed choice about contraception should emphasize the advantages and disadvantages, the health benefits, risks and side-effects, and should enable comparison of various contraceptive methods. Censoring, withholding or intentionally misrepresenting information about contraception can put health and basic human rights in jeopardy*.

## EFFECTIVENESS AND OTHER METHOD CHARACTERISTICS

Research consistently shows women believe effectiveness is one of the most important factors when choosing a contraceptive method^28^^-^^30^; in many studies, effectiveness is mentioned as most important by a clear majority of women.^1^^,^^17^^,^^31^^-^^33^ Issues of side effects, the ability to use the method covertly, or the ability to control initiation and/or cessation of use are also important to women and should always be discussed. In some situations, they may “trump” effectiveness for certain women. However, to ensure that decision making is based on accurate information, effectiveness should be the fundamental starting point in describing methods for women seeking contraceptive services.

Effectiveness is one of the most important factors, and indeed often *the* most important factor, when women choose a method.

Given the evidence of women’s stated preferences, the 20-fold increased protection from unintended pregnancy that LARCs can provide, as well as the reality that approximately 40% of unintended pregnancies end in abortion,^34^ proactive counseling using the WHO tiers is simple, common sense. Among the many who have come to agree with counseling about the most effective methods first are the American Academy of Pediatricians (AAP), the American College of Obstetricians and Gynecologists, and the Centers for Disease Control and Prevention.^35^^-^^37^ The AAP 2014 policy statement on contraception for adolescents encourages providers to counsel by “discussing the most effective contraceptive methods first.”^35^ Internationally, the highly regarded “Balanced Counseling Strategy,” developed by the Population Council, also makes use of WHO-tiered counseling. Users of this popular counseling tool first help the client rule out certain classes of methods, then are guided by the strategy’s algorithm to: (1) visually present the remaining methods in order of effectiveness, (2) fully explain the concept of effectiveness, and (3) counsel the client beginning with the most effective methods.^38^

## COMPREHENSIVE COUNSELING

The WHO contraceptive effectiveness tiers are only one piece in a larger framework of comprehensive counseling that must include private, client-centered conversation about a woman’s reproductive needs and desires. WHO-tiered counseling should not be equated with directive counseling, and it does not assume that a woman *should* choose a LARC. Nor does it dismiss the side effects or other characteristics of any method. Rather, it should help a client put those characteristics in a perspective that includes contraceptive effectiveness and pregnancy risk.

**FIGURE 2:**
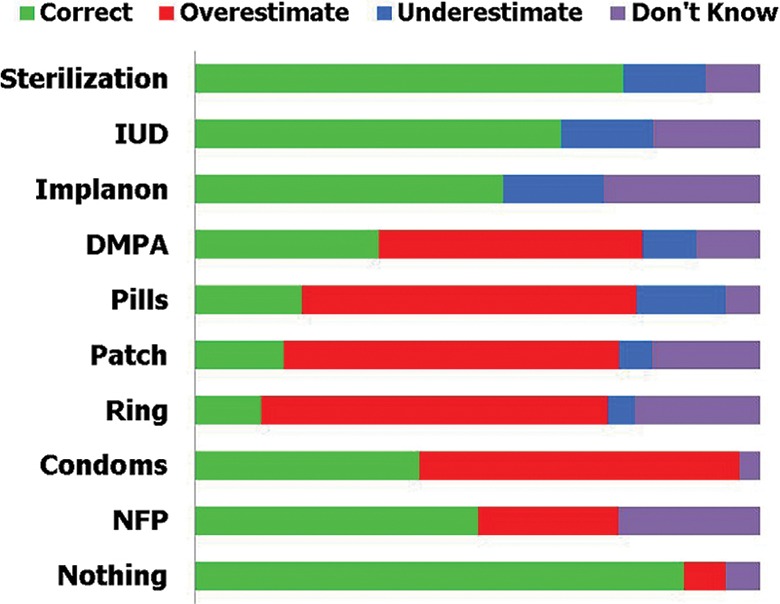
Knowledge of Contraceptive Effectiveness Among Participants in the St. Louis CHOICE Study (N=4,144) Abbreviations: DMPA, depot medroxyprogesterone acetate; IUD, intrauterine device; NFP, natural family planning. Source: Reprinted from Eisenberg et al.,^2^ with permission from Elsevier.

Conveying that risk, i.e., an understanding of the relative effectiveness of different methods, is challenging. Often, the absolute and relative effectiveness of different contraceptive methods are misunderstood by clients. For example, at enrollment, women in the St. Louis CHOICE study significantly overestimated the effectiveness of various non-LARC methods, while significantly underestimating the effectiveness of LARCs (Figure 2).^2^ Such misconceptions are widespread, leading to “misinformed choice” among women unless these misunderstandings are corrected by providers. While “misinformed choice” does not rise to the level of coercion, we agree with WHO^3^ that programs that do not fully and comprehensively educate women about method effectiveness and ensure that clients understand the differences between methods are not rights-based.

Providers have a responsibility to educate women about method effectiveness to help avoid “misinformed choice.”

## NO LARCS IS NO EXCUSE

Another reason that providers may deemphasize LARCs during counseling is that long-acting methods may not be available or affordable in their setting. When this happens, we fail women by providing both counseling *and* service delivery that are not rights-based. Lack of access to LARCs remains a major problem both in the developed and the developing worlds. In low-resource regions, LARCs may not be available at all, or, where available in theory, may be unaffordable or impossible to access due to provider bias, outdated knowledge, or lack of training.^39^ Thus, millions of women are not receiving rights-based provision of family planning because they lack either information about and/or access to the full range of modern methods, and especially to LARCs. Our priority is increasing access to WHO tier-1 methods themselves, along with accurate education about their advantages and disadvantages.

## CONCLUSION

For societies to reap the many benefits of family planning, both at the individual and macro levels, all methods of family planning, reversible *and* permanent, should be widely—indeed universally—available. Provision of these methods must include free choice, discontinuation on demand, and comprehensive counseling that proactively focuses on the WHO tiers of effectiveness. Until then, we are failing to accurately inform women with rights-based family planning programs.

## Notes

**Peer Reviewed**
